# Eating disorders among people with and without type 1 diabetes: incidence and treatment in a nationwide population-based cohort

**DOI:** 10.1007/s00125-024-06346-7

**Published:** 2025-01-04

**Authors:** Leon Hirvelä, Jari Haukka, Anna Keski-Rahkonen, Pyry N. Sipilä

**Affiliations:** Department of Public Health, University of Helsinki, Helsinki, Finland

**Keywords:** Eating disorders, Hospital treatment, Incidence, Psychotropic medications, Type 1 diabetes

## Abstract

**Aims/hypothesis:**

Eating disorders are over-represented in type 1 diabetes and are associated with an increased risk of complications, but it is unclear whether type 1 diabetes affects the treatment of eating disorders. We assessed incidence and treatment of eating disorders in a nationwide sample of individuals with type 1 diabetes and diabetes-free control individuals.

**Methods:**

Our study comprised 11,055 individuals aged <30 who had been diagnosed with type 1 diabetes in 1998–2010, and 11,055 diabetes-free control individuals matched for age, sex and hospital district. We ascertained incidence of eating disorders from hospital records using Poisson regression. Eating disorder treatment was assessed by new prescriptions for psychotropic medications and hospital treatment for eating disorders.

**Results:**

During a mean follow-up of 13.1 years, there were 175 incident cases of eating disorders among individuals with type 1 diabetes and 75 among the control individuals (adjusted incidence rate ratio 2.35; 95% CI 1.80, 3.09). The prescription of psychotropic medications was similar among eating disorder patients with and without type 1 diabetes. However, those with type 1 diabetes received outpatient hospital treatment for their eating disorder less often than those without diabetes (mean 3.32 vs 5.33 outpatient care visits per year [adjusted difference 1.24; 95% CI 0.39, 2.08]).

**Conclusions/interpretation:**

People with type 1 diabetes are more likely to be diagnosed with eating disorders than their diabetes-free peers. However, they receive less outpatient hospital treatment for their eating disorders despite their greater risk for major adverse health outcomes. These findings emphasise the need for targeted eating disorder treatment for people with type 1 diabetes.

**Graphical Abstract:**

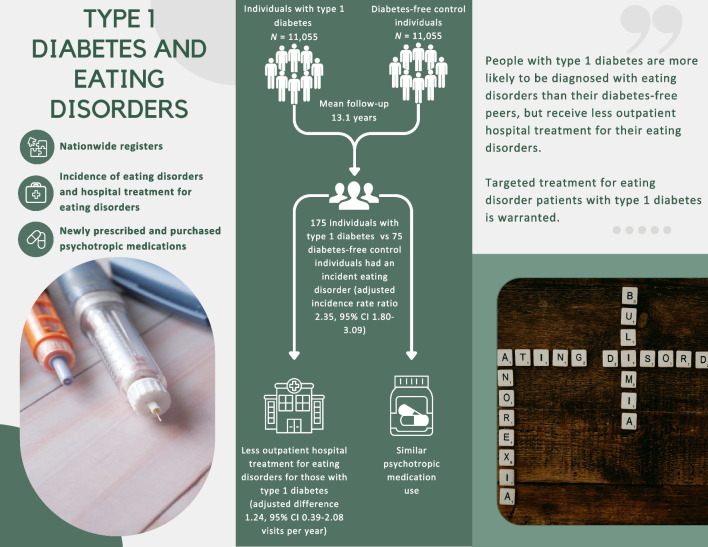

**Supplementary Information:**

The online version contains peer-reviewed but unedited supplementary material available at 10.1007/s00125-024-06346-7.


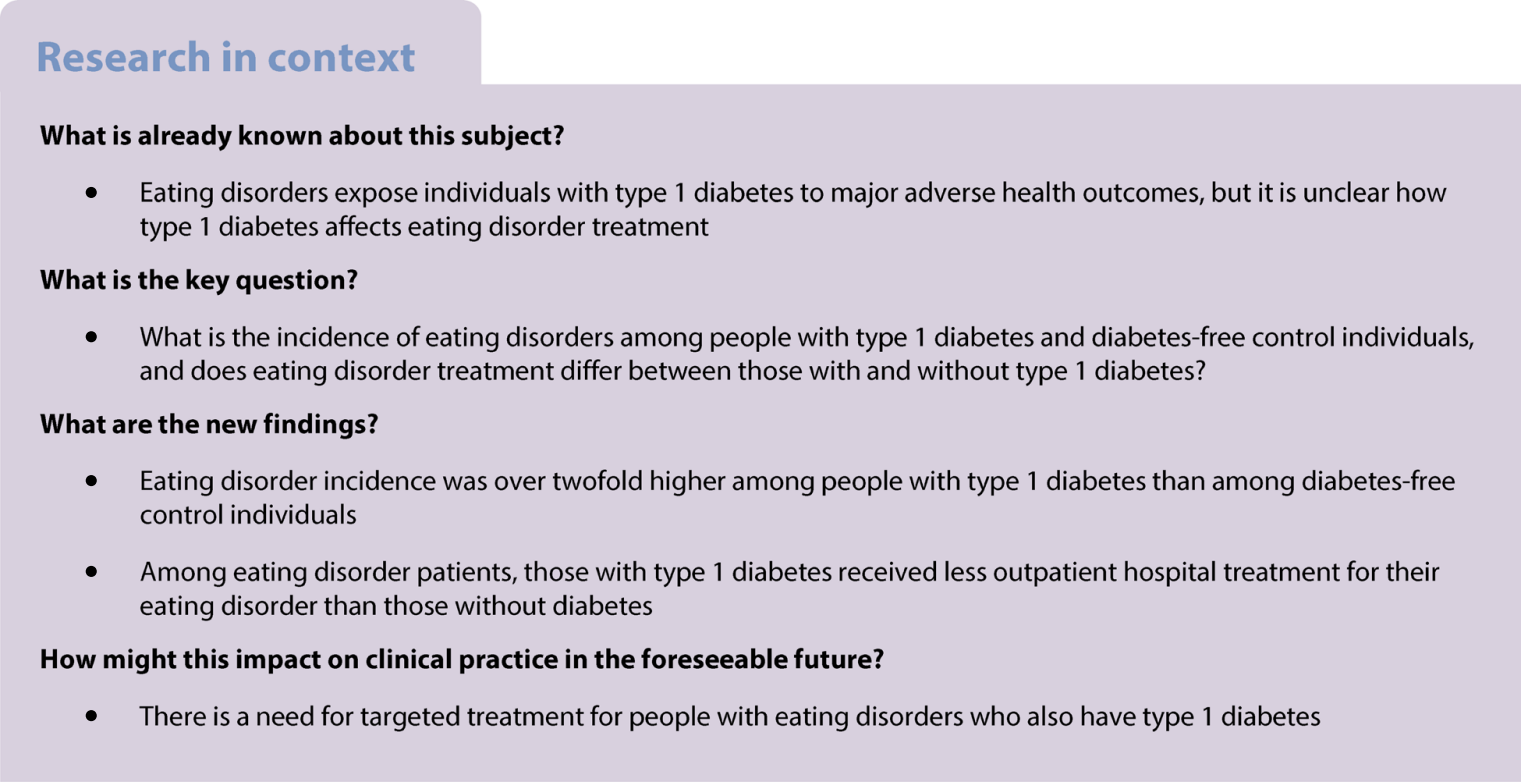


## Introduction

Eating disorders are common with an estimated lifetime prevalence of up to 10.5% among young adults [1, 2]. Their incidence and prevalence are at least twofold in people with type 1 diabetes when compared with diabetes-free control individuals [3–5]. Underlying reasons are most likely diverse [6–10], including psychological aspects specific to type 1 diabetes such as illness perceptions, coping strategies and insulin beliefs [8, 10]. Although sound, diabetes-specific, risk factor studies are lacking, the complex needs of type 1 diabetes self-care, such as continuously monitoring blood glucose levels, diabetes distress and psychological comorbidity, such as depression and anxiety, might also contribute.

Insulin restriction is a hallmark and a unique compensatory behaviour for eating disorder patients with type 1 diabetes, differentiating them from those eating disorder patients who do not have type 1 diabetes [11]. Eating disorders among individuals with type 1 diabetes are accompanied by major increases in diabetes-related morbidity, including nephropathy, retinopathy, neuropathy [12–16] and ketoacidosis, which is potentially fatal [15, 17]. Mortality rate is also increased [13, 17, 18].

Current guidelines for eating disorder treatment include nutritional rehabilitation and weight restoration, psychotherapy, family-based treatment and, to a lesser extent, psychotropic medications such as antipsychotics, antidepressants and anxiolytics [19, 20]. Despite the marginal role of psychotropic medications in the guidelines for eating disorder treatment, these medications are frequently used among eating disorder patients [21–23]. Patients with co-occurring type 1 diabetes seem to benefit less from these treatment options, and adherence issues and high dropout rates are common [24–26]. However, these findings are based on case series [25], quasi-experimental designs [26–29] and only one small randomised controlled trial [30]. There is a lack of evidence-based treatments for eating disorders among type 1 diabetes patients [31], which might contribute to undertreatment.

There is limited research on the differences in psychotropic medication use between eating disorder patients with and without type 1 diabetes. The use of psychotropic medications is generally more common in individuals with type 1 diabetes compared to those without diabetes [32], possibly due to the increased prevalence of anxiety and depression symptoms [3, 4]. Eating disorder patients with type 1 diabetes tend to have poorer responses to standard eating disorder treatments [24–26], which might increase their likelihood of receiving psychotropic medications. On the other hand, clinicians may be more cautious in prescribing certain psychotropic medications, such as antipsychotics, to patients with type 1 diabetes due to their potential adverse metabolic effects [33]. Yet, to our knowledge, no large-scale epidemiological studies to date have assessed whether eating disorder patients with co-occurring type 1 diabetes receive less treatment for their eating disorders than diabetes-free control patients. Moreover, despite Finland’s high prevalence of eating disorders [1] and the highest incidence of type 1 diabetes globally [34], we are aware of no studies investigating the incidence and treatment of eating disorders among Finnish individuals with type 1 diabetes.

We aimed to assess the incidence of hospital-treated eating disorders in people with type 1 diabetes compared with that among diabetes-free control individuals in a nationwide setting, with a follow-up for up to 20 years. Further, among people with eating disorders, we aimed to assess the course of their treatment, and whether this treatment differed between those with type 1 diabetes compared to those without diabetes. We assessed eating disorder treatment by new prescriptions for psychotropic medications and in- and outpatient hospital treatment for eating disorders.

## Methods

### Study samples

This study is based on the CARING (CAncer Risk and INsulin analoGues) project [35], which consists of a cohort of participants with diabetes and diabetes-free control participants. In the project, data on prescription medication purchases and reimbursements were provided by the Social Insurance Institution of Finland [36]. Patients with diabetes were defined as insulin users or users of blood glucose-lowering drugs excluding insulin, i.e. individuals who had purchased and received reimbursement for at least one insulin prescription (the Anatomical Therapeutic Chemical [ATC] code A10A) or blood glucose-lowering drugs excluding insulin prescription (ATC code A10B) between 1 January 1997 and 31 December 2010. All identified insulin users, as well as a 50% random sample of users of blood glucose-lowering drugs excluding insulin, during this period in Finland were included in the study. The year 1996 was used as an initial wash-in period to guarantee that no individual in the original population had a prescription for a glucose-lowering drug within the 12 months prior to the start of the study period. The diabetes-free control individuals were individually matched to the diabetes group by age (±1 year), sex (as recorded in the Finnish Population Information System maintained by the Digital and Population Data Services Agency), place of residence (hospital district) and start of the follow-up (the exact date). The size of the original population was 398,708 individuals, comprising 199,354 individuals with diabetes and 199,354 control individuals. The study plan was approved by the ethics committee of the Faculty of Medicine, University of Helsinki, on 17 January 2012.

**Fig. 1:**
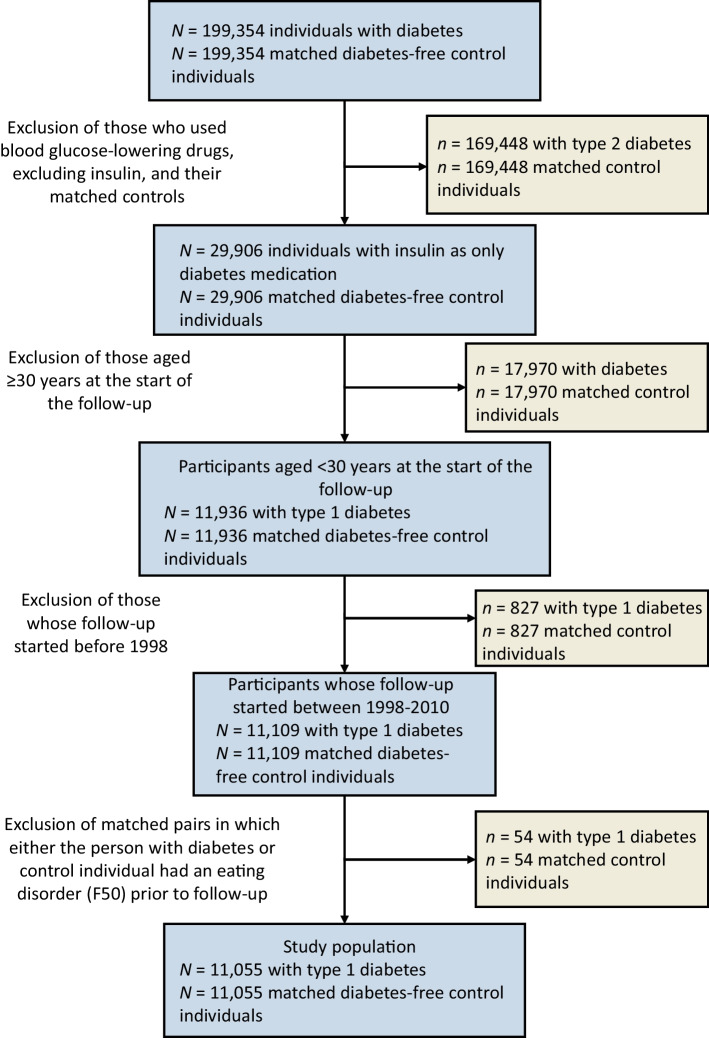
Flowchart describing study sample selection

For the current study, we selected from the CARING project participants with type 1 diabetes and their diabetes-free control participants. We defined patients with type 1 diabetes as individuals with only insulin as glucose-lowering medication in the start of the follow-up, and who were aged less than 30 years [37, 38]. To assess only newly diagnosed type 1 diabetes patients, we excluded all participants who had purchased glucose-lowering medications during the initial wash-in period in 1996 or during 1997. Thus, the dates for first insulin prescription ranged from 1 January 1998 to 31 December 2010, and the follow-up for eating disorders started on that day. After excluding all individuals with an eating disorder prior to follow-up, our analysis comprised 11,055 individuals with type 1 diabetes and 11,055 diabetes-free control individuals, who were followed-up for incident eating disorders until the date of first eating disorder diagnosis, death, or 31 December 2017, whichever occurred first. See Fig. 1 for a flowchart.

### Eating disorder cases

Information on new eating disorder diagnoses during the follow-up was gathered from the Care Register for Health Care, which is maintained by the Finnish Institute for Health and Welfare [39] and comprises virtually all inpatient and outpatient hospital care in Finland. Records from primary healthcare are not included in the Care Register. We ascertained eating disorders using the following ICD-10 codes: F50.0 (anorexia nervosa), F50.1 (atypical anorexia nervosa), F50.2 (bulimia nervosa), F50.3 (atypical bulimia nervosa), F50.4 (overeating associated with other psychological disturbances), F50.5 (vomiting associated with other psychological disturbances), F50.8 (other eating disorders) and F50.9 (eating disorder, unspecified). Both primary and secondary diagnoses were included.

### Psychotropic medications and hospital treatment

We obtained information on psychotropic medication use after an eating disorder diagnosis using reimbursement data from the Social Insurance Institution of Finland. To assess eating disorder treatment-associated psychotropic medication use, we excluded from these analyses those who had used these medications before eating disorder diagnosis. We included the following ATC codes (drug categories): N05A (antipsychotics), N06A (antidepressants) and N05B (anxiolytics). To assess genuine antipsychotic use, from the antipsychotics group, we excluded lithium (N05AN01), which is a mood stabiliser, and prochlorperazine (N05AB04), which is mostly used as an antiemetic [23].

Hospital treatment was assessed as outpatient visits and inpatient care days per year after the diagnosis of an eating disorder. Data were obtained from the Care Register for Health Care. Our primary interest was hospital treatment for eating disorders. We also assessed hospital treatment for other mental health diagnoses, using ICD-10 codes F10 (mental and behavioural disorders due to use of alcohol), F32–F34.1 (depressive episode, recurrent depressive disorder, cyclothymia and dysthymia), F41 (other anxiety disorders), F42 (obsessive–compulsive disorder), F43.1 (post-traumatic stress disorder), F60 (specific personality disorders) and F84 (pervasive developmental disorders). Last, we assessed hospital treatment for any cause, including somatic causes. Diagnoses were retrieved from both primary and secondary diagnoses on each inpatient care stay and outpatient care visit.

### Covariates

Data on socioeconomic status and place of residence were obtained from Statistics Finland [40]. Socioeconomic status was classified according to the official classification of Statistics Finland as follows: upper-level employees, self-employed, lower-level employees, manual workers, students, pensioners and others [40].

For those individuals who were younger than 15 years at the start of the follow-up, the individual’s socioeconomic group was marked the same as their guardian’s. Data on socioeconomic status were available only from the time point when the individual entered the follow-up. Place of residence was obtained at the level of accuracy of the individual’s healthcare district in the start of the follow-up, comprising 21 geographical areas in Finland. For some analyses, we grouped these to two groups: secondary healthcare district vs tertiary healthcare district, the latter representing more specialised healthcare and urban environment.

### Statistical analysis

We report baseline characteristics as *n* (%) for categorical variables and as mean (SD) and median (IQR) for continuous variables. We used Poisson regression to model incidence and incidence rate ratios (IRRs) with 95% CIs. First, we assessed incidence (per 100,000 person-years) and IRR of eating disorders among individuals with type 1 diabetes vs control participants. We performed both unadjusted and multiple-adjusted models. The unadjusted models estimate crude incidence and IRRs, i.e. we did not adjust them for any of the available variables. We adjusted the multiple-adjusted models for age, sex, date of the start of follow-up, socioeconomic status and place of residence (at the level of accuracy of the original 21 healthcare districts). Additional analyses assessing sex, socioeconomic status or some other variable instead of diabetes status were adjusted for diabetes status instead of the exposure variable in question. Second, among all eating disorder cases detected, we assessed incidence (per 1000 person-years) and IRR of psychotropic medication use for individuals with type 1 diabetes vs individuals without diabetes. We investigated only those cases where there was no prior use of the drug group in question before diagnosis of an eating disorder. Finally, we assessed associations of diabetes status, sex and place of residence with means and 95% CIs of outpatient care visits and inpatient care days per year using linear regression. We adjusted these analyses in the same manner as the incidence analyses. We report all confidence intervals at the 95% level and all *p* values are two-sided. We carried out all analyses with R version 4.3.0.

## Results

Female participants comprised 47.5% (*n*=10,510) of our sample (Table 1). Mean age (SD) was 14.1 (8.3) years at the start of the eating disorder follow-up.

**Table 1:** Baseline characteristics of study population

Characteristic	Overall	Type 1 diabetes group	Diabetes-free control group
	*N*=22,110	*N*=11,055	*N*=11,055
Female sex	10,510 (47.5)	5255 (47.5)	5255 (47.5)
Age			
Mean ±SD	14.1±8.3	14.1±8.3	14.1±8.3
Median [IQR]	12.3 [7.2–21.1]	12.3 [7.2–21.1]	12.3 [7.2–21.1]
Age groups			
0 – <10	8470 (38.3)	4235 (38.3)	4235 (38.3)
10 – <15	4986 (22.6)	2493 (22.6)	2493 (22.6)
15 – <20	2670 (12.1)	1335 (12.1)	1335 (12.1)
20 – <25	2374 (10.7)	1187 (10.7)	1187 (10.7)
25 – <30	3610 (16.3)	1805 (16.3)	1805 (16.3)
Socioeconomic status^a^			
Upper-level employees	3616 (16.4)	1820 (16.5)	1796 (16.2)
Self-employed	1583 (7.2)	775 (7.0)	808 (7.3)
Lower-level employees	4816 (21.8)	2394 (21.7)	2422 (21.9)
Manual workers	5676 (25.7)	2898 (26.2)	2778 (25.1)
Students	2615 (11.8)	1275 (11.5)	1340 (12.1)
Pensioners	337 (1.5)	187 (1.7)	150 (1.4)
Others	3467 (15.7)	1706 (15.4)	1761 (15.9)
Place of residence			
Secondary healthcare district	9894 (44.7)	4947 (44.7)	4947 (44.7)
Tertiary healthcare district	12,216 (55.3)	6108 (55.3)	6108 (55.3)

Data are *n* (%), except where otherwise indicated

^a^For those participants who were younger than 15 years at baseline, the socioeconomic status was classified the same as their guardian’s

During a mean (SD) follow-up of 13.1 (3.8) years, there were 175 incident cases of eating disorders among those with type 1 diabetes and 75 among the control participants, which yielded an adjusted incidence rate ratio (aIRR) of 2.35 (95% CI 1.80, 3.09) (Table 2). Eating disorders were more common in women than men (aIRR 8.74; 95% CI 5.99, 12.75). We found no interaction between sex and diabetes status on the incidence of eating disorders (*p* for interaction 0.76). Due to the small number of eating disorder cases in some of the diagnostic subgroups, for analysis of eating disorders subtypes, we pooled together: (1) all the anorexia subtypes, (2) all the bulimia subtypes, and (3) the residual eating disorder subtypes (electronic supplementary material [ESM] Table 1). We found no differences in the distribution of eating disorder subtypes between those with type 1 diabetes and those without diabetes in these pooled groups (*p* for χ^2^ test 0.11).

**Table 2:** Association of type 1 diabetes and participant characteristics with the incidence of eating disorders

Variable	Group	Person-years	Eating disorder events	Rate (95% CI) per 100,000 person-years	IRR (95% CI)	aIRR (95% CI)
Diabetes status	No diabetes	146,000	75	51.5 (40.5, 64.6)	1.00 (ref)	1.00 (ref)
	Type 1 diabetes	144,000	175	121.5 (104.2, 140.9)	2.36 (1.80, 3.09)	2.35 (1.80, 3.09)
Sex	Male	154,000	31	20.2 (13.7, 28.6)	1.00 (ref)	1.00 (ref)
	Female	136,000	219	161.3 (140.6, 184.1)	8.00 (5.50, 11.66)	8.74 (5.99, 12.75)
Time-varying age (years)	0 – <10	74,000	37	50.1 (35.3, 69.0)	1.00 (ref)	1.00 (ref)
	10 – <15	64,000	130	204.0 (170.5, 242.3)	4.08 (2.83, 5.87)	1.45 (1.08, 1.93)
	15 – <20	49,000	43	87.3 (63.2, 117.6)	1.74 (1.12, 2.71)	0.90 (0.57, 1.41)
	20 – <25	37,000	17	45.7 (26.6, 73.1)	0.91 (0.51, 1.62)	0.37 (0.20, 0.69)
	25 – <30	41,000	19	46.3 (27.9, 72.3)	0.92 (0.53, 1.61)	0.32 (0.20, 0.53)
	≥30	n/a	<5	n/a	n/a	n/a
Place of residence	Secondary healthcare district	130,000	79	60.9 (48.2, 75.8)	1.00 (ref)	1.00 (ref)
	Tertiary healthcare district	160,000	171	107.0 (91.6, 124.3)	1.76 (1.35, 2.30)	1.70 (1.30, 2.22)

Crude IRRs were not adjusted for anything. The aIRRs were adjusted for diabetes status, sex, age, start of follow-up, socioeconomic status and place of residence

n/a, not available (data for groups with <5 participants are not shown to protect the privacy of the participants)

The use of psychotropic medications among the detected eating disorder cases was substantial (Table 3). The amounts (%) of new drug users among the 250 eating disorder cases were as follows: any psychotropic medication (comprising antipsychotics, antidepressants and anxiolytics) 154 (61.6%), antipsychotics 44 (17.6%), antidepressants 96 (38.4%), and anxiolytics 33 (13.2%). We found no differences in the use of psychotropic medications between those eating disorder patients with type 1 diabetes and those without diabetes: aIRR (95% CI) for any psychotropic medication 1.28 (0.85, 1.93), for antipsychotics 0.63 (0.32, 1.23), for antidepressants 1.25 (0.73, 2.14) and for anxiolytics 0.82 (0.35, 1.90).

**Table 3:** Use of newly prescribed psychotropic medications among eating disorder patients per 1000 person-years

Psychotropic medication	Events (%)	Person-years	Rate (95% CI) per 1000 person-years	IRR (95% CI)	aIRR (95% CI)
Any psychotropic medication					
Diabetes status					
No diabetes	51 (68.0)	259	196.8 (146.5, 258.7)	1.00 (ref)	1.00 (ref)
Type 1 diabetes	103 (58.9)	562	183.4 (149.7, 222.4)	0.93 (0.67, 1.30)	1.28 (0.85, 1.93)
Sex					
Male	16 (51.6)	125	127.8 (73.0, 207.5)	1.00 (ref)	1.00 (ref)
Female	138 (63.0)	696	198.4 (166.7, 234.4)	1.55 (0.93, 2.61)	2.05 (1.14, 3.68)
Place of residence					
Tertiary healthcare district	103 (60.2)	620	166.0 (135.5, 201.3)	1.00 (ref)	1.00 (ref)
Secondary healthcare district	51 (64.6)	200	254.5 (189.5, 334.7)	1.53 (1.10, 2.14)	2.24 (1.52, 3.32)
Antipsychotics					
Diabetes status					
No diabetes	18 (24.0)	398	45.2 (26.8, 71.5)	1.00 (ref)	1.00 (ref)
Type 1 diabetes	26 (14.9)	996	26.1 (17.1, 38.3)	0.58 (0.32, 1.05)	0.63 (0.32, 1.23)
Sex					
Male	<5	n/a	n/a	n/a	n/a
Female	40 (n/a)	1209	33.1 (23.6, 45.0)	n/a	n/a
Place of residence					
Tertiary healthcare district	26 (15.2)	1,024	25.4 (16.6, 37.2)	1.00 (ref)	1.00 (ref)
Secondary healthcare district	18 (22.8)	370	48.6 (28.8, 76.9)	1.92 (1.05, 3.49)	2.24 (1.18, 4.25)
Antidepressants					
Diabetes status					
No diabetes	32 (42.7)	270	118.3 (80.9, 167.0)	1.00 (ref)	1.00 (ref)
Type 1 diabetes	64 (36.6)	537	119.1 (91.7, 152.1)	1.01 (0.66, 1.54)	1.25 (0.73, 2.14)
Sex					
Male	9 (29.0)	131	68.7 (31.4, 130.5)	1.00 (ref)	1.00 (ref)
Female	87 (39.7)	677	128.5 (103.0, 158.6)	1.87 (0.94, 3.72)	2.69 (1.24, 5.83)
Place of residence					
Tertiary healthcare district	68 (39.8)	617	110.3 (85.6, 139.8)	1.00 (ref)	1.00 (ref)
Secondary healthcare district	28 (35.4)	191	146.4 (97.3, 211.6)	1.33 (0.86, 2.06)	1.43 (0.85, 2.40)
Anxiolytics					
Diabetes status					
No diabetes	11 (14.7)	463	23.8 (11.9, 42.5)	1.00 (ref)	1.00 (ref)
Type 1 diabetes	22 (12.6)	1028	21.4 (13.4, 32.4)	0.90 (0.44, 1.86)	0.82 (0.35, 1.90)
Sex					
Male	<5	n/a	n/a	n/a	n/a
Female	31 (n/a)	1297	23.9 (16.2, 33.9)	n/a	n/a
Place of residence					
Tertiary healthcare district	22 (12.9)	1079	20.4 (12.8, 30.9)	1.00 (ref)	1.00 (ref)
Secondary healthcare district	11 (13.9)	412	26.7 (13.3, 47.8)	1.31 (0.64, 2.70)	1.56 (0.71, 3.42)

Crude IRRs were not adjusted for anything. aIRRs were adjusted for age at baseline, sex, diabetes status, socioeconomic status and place of residence

Only those cases were investigated who had no prior use of the drug group in question before diagnosis of an eating disorder

‘Any psychotropic medication’ includes use of either antipsychotics, antidepressants or anxiolytics, i.e. the eating disorder patient was included in this category when purchasing any of these psychotropic medications for the first time

n/a, not available (data for groups with <5 participants are not shown to protect the privacy of the participants)

Eating disorder patients with type 1 diabetes received less outpatient hospital treatment for their eating disorders than those without diabetes (mean 3.32 vs 5.33 outpatient care visits per year [adjusted difference 1.24; 95% CI 0.39, 2.08]) (Table 4). However, we did not detect any difference in the adjusted model for inpatient hospital treatment for eating disorders (mean 2.41 vs 3.69 inpatient care days per year [adjusted difference 0.17; 95% CI −0.77, 1.11]). Male participants received less both outpatient (mean 1.95 vs 4.21 outpatient care visits per year) and inpatient (mean 0.68 vs 3.10 inpatient care days per year) hospital treatment for their eating disorders than female participants (for the adjusted difference and 95% CIs, see Table 4). As for treatment for mental disorders other than eating disorders, those eating disorder patients with type 1 diabetes received more outpatient hospital treatment (mean 2.43 vs 1.42 outpatient care visits per year) but not inpatient hospital treatment (mean 1.69 vs 0.89 inpatient care days per year) than those without diabetes. Finally, those eating disorder patients with type 1 diabetes received hospital treatment for any cause more frequently than those without diabetes in terms of outpatient care (mean 10.40 vs 6.79 outpatient care visits per year) but not inpatient care (mean 4.72 vs 4.57 inpatient care days per year). The adjusted differences and 95% CIs are reported in Table 4.

**Table 4:** Hospital treatment among eating disorder patients

	Inpatient hospital care				Outpatient hospital care			
	Median	Mean (SD)	β (95% CI)	adjusted β (95% CI)	Median	Mean (SD)	β (95% CI)	adjusted β (95% CI)
Inpatient/outpatient hospital care for eating disorders	0.00	2.80 (9.83)			0.86	3.93 (8.66)		
Diabetes status								
No diabetes	0.00	3.69 (11.51)	(ref)	(ref)	1.53	5.33 (8.65)	(ref)	(ref)
Type 1 diabetes	0.00	2.41 (8.97)	−1.27 (−2.23, −0.32)	−0.17 (−1.11, 0.77)	0.69	3.32 (8.60)	−2.00 (−2.84, −1.16)	−1.24 (−2.08, −0.39)
Sex								
Male	0.00	0.68 (2.67)	(ref)	(ref)	0.72	1.95 (5.17)	(ref)	(ref)
Female	0.00	3.10 (10.41)	2.42 (1.08, 3.76)	3.08 (1.75, 4.41)	1.04	4.21 (9.00)	2.25 (1.07, 3.43)	4.00 (2.80, 5.19)
Place of residence								
Tertiary healthcare district	0.00	2.61 (8.79)	(ref)	(ref)	1.21	4.18 (8.77)	(ref)	(ref)
Secondary healthcare district	0.00	3.23 (11.86)	0.61 (−0.34, 1.57)	1.05 (0.06, 2.03)	0.31	3.36 (8.38)	−0.82 (−1.66, 0.02)	−0.63 (−1.50, 0.23)
Inpatient/outpatient hospital care for other mental disorders	0.00	1.44 (7.26)			0.09	2.12 (7.79)		
Diabetes status								
No diabetes	0.00	0.89 (10.18)	(ref)	(ref)	0.00	1.42 (3.60)	(ref)	(ref)
Type 1 diabetes	0.00	1.69 (5.52)	0.80 (0.10, 1.51)	0.61 (−0.14, 1.35)	0.25	2.43 (9.01)	1.00 (0.25, 1.76)	0.81 (0.00, 1.61)
Sex								
Male	0.00	0.86 (3.55)	(ref)	(ref)	0.05	1.11 (2.80)	(ref)	(ref)
Female	0.00	1.53 (7.63)	0.67 (−0.33, 1.66)	0.10 (−0.95, 1.15)	0.09	2.26 (8.23)	1.15 (0.09, 2.22)	0.67 (−0.47, 1.81)
Place of residence								
Tertiary healthcare district	0.00	1.30 (5.06)	(ref)	(ref)	0.10	2.16 (8.90)	(ref)	(ref)
Secondary healthcare district	0.00	1.76 (10.69)	0.46 (−0.24, 1.17)	0.38 (−0.36, 1.12)	0.08	2.04 (4.28)	−0.13 (−0.88, 0.63)	−0.03 (−0.83, 0.76)
Any inpatient/outpatient hospital care	0.35	4.68 (13.02)			5.79	9.31 (13.76)		
Diabetes status								
No diabetes	0.00	4.57 (16.88)	(ref)	(ref)	2.56	6.79 (9.45)	(ref)	(ref)
Type 1 diabetes	0.58	4.72 (10.92)	0.15 (−1.11, 1.42)	1.07 (−0.24, 2.37)	7.83	10.40 (15.14)	3.61 (2.28, 4.94)	4.58 (3.19, 5.96)
Sex								
Male	0.00	1.85 (4.35)	(ref)	(ref)	3.75	5.92 (6.42)	(ref)	(ref)
Female	0.44	5.07 (13.75)	3.22 (1.45, 5.00)	3.34 (1.50, 5.19)	6.22	9.78 (14.43)	3.86 (1.98, 5.73)	5.32 (3.36, 7.27)
Place of residence								
Tertiary healthcare district	0.43	4.38 (10.63)	(ref)	(ref)	5.97	9.64 (15.10)	(ref)	(ref)
Secondary healthcare district	0.21	5.35 (17.26)	0.97 (−0.30, 2.23)	1.47 (0.15, 2.79)	5.70	8.55 (10.07)	−1.08 (−2.42, 0.25)	−0.08 (−1.46, 1.29)

Crude βs were not adjusted for anything. Adjusted βs were adjusted for age at baseline, sex, diabetes status, socioeconomic status and place of residence

‘Any inpatient hospital care’ and ‘any outpatient hospital care’ include both psychiatric care and care for somatic causes

## Discussion

In our nationwide cohort study, we found that individuals with type 1 diabetes had more than a twofold higher incidence of eating disorders than diabetes-free control individuals. Among the 250 eating disorder cases detected, over 60% received newly prescribed psychotropic medications, with no differences observed between those with vs without type 1 diabetes. However, those with type 1 diabetes received less outpatient hospital treatment for their eating disorders than those without diabetes.

To our knowledge, this is the first nationwide study to assess differences in hospital treatment for eating disorders between people with and without type 1 diabetes. Our findings indicating that eating disorders in patients with type 1 diabetes are undertreated relative to those without diabetes are in line with previous small, single-centred studies, which have also reported less intensive treatment for those eating disorder patients with type 1 diabetes [25, 26]. These findings are concerning, as eating disorders are associated with increased morbidity and mortality rates among type 1 diabetes patients [12–18]. Thus, eating disorders among people with type 1 diabetes should be treated more intensively, not less intensively, than eating disorders in general. Treatment outcomes are also poorer among those eating disorder patients who also have type 1 diabetes when compared with those who do not [24–26], which further highlights the need for rigorous treatment. Future studies should study associations between eating disorder treatment and diabetes-related outcomes, such as glycaemic control and complications.

Reasons for the undertreatment of eating disorders among those with type 1 diabetes have been assessed previously. Adherence issues are common [25], and dropout rates are high [26]. High dropout rates have been associated with the patients’ lower motivation and the perceived intensity of the eating disorder. It is possible that the eating disorders among people with type 1 diabetes could be less severe than the eating disorders of those with no diabetes. Although the difference was not statistically significant, in our study a slightly higher proportion of eating disorders seemed to be diagnosed as ‘other eating disorder’ among type 1 diabetes patients than among diabetes-free control patients. However, the diagnosis of ‘other eating disorder’ is not necessarily less severe than anorexia nervosa or bulimia nervosa among those with type 1 diabetes; it could also reflect atypical features of eating disorders, such as insulin omission. We could not directly assess the severity of eating disorders, but earlier evidence indicates that the psychiatric and medical characteristics of eating disorders do not appear to differ between those with or without type 1 diabetes [26]. It is also possible that some eating disorder-related outpatient visits of those with type 1 diabetes could have been recorded with only a diabetes diagnosis. However, as hospital treatment for eating disorders is given mostly in highly specialised psychiatric eating disorder units in Finland, it is unlikely that the clinicians would not have recorded any eating disorder diagnosis for these hospital visits. Other possible reasons for undertreatment of eating disorders among type 1 diabetes patients include the lack of evidence-based treatments available for this group [31], the complexity of treating co-occurring eating disorders and type 1 diabetes and lack of healthcare personnel with adequate knowledge about both eating disorders and type 1 diabetes. Qualitative research has shed light on the care experiences of patients with co-occurring eating disorders and type 1 diabetes and indicated a need for multidisciplinary collaboration and professionals who understand both disorders [41].

We are not aware of previous studies assessing the use of psychotropic medications among eating disorder patients with type 1 diabetes, let alone comparisons with eating disorder patients without diabetes. Our findings of a substantial use of these medications, with no differences between type 1 diabetes and diabetes-free eating disorder patients, is noteworthy. Despite eating disorder patients with type 1 diabetes responding less well to eating disorder treatment [25, 26], they appear not to be treated differently with psychotropic medications compared with diabetes-free patients, even though type 1 diabetes comes with an elevated risk for anxiety and depression [3, 4]. We suspect that, because of the timeframe for consultation, there are limits to how psychiatric problems can be addressed when there are additional complicating factors such as co-occurring type 1 diabetes. In addition, clinicians might be in general more cautious in prescribing psychotropic medications to those with type 1 diabetes due to their adverse metabolic effects. Further, despite these effects, which might occur even independent of weight gain [33], 15% of type 1 diabetes patients with an eating disorder had used antipsychotics in the present study, which might contribute to worse metabolic balance and increased morbidity and mortality rates [42]. Future studies should assess the outcomes and safety of antipsychotic medication use among eating disorder patients with type 1 diabetes.

Our results of over twofold higher incidence of eating disorders among people with type 1 diabetes compared to diabetes-free individuals complement previous similar findings [3, 4]. Dybdal et al used hospital registers to assess eating disorder incidence with somewhat similar findings to ours (aIRR 2.35 [95% CI 1.80, 3.09] in our study vs HR 2.02 [95% CI 1.54, 2.64] in girls and 3.73 [95% CI 1.71, 8.11] in boys in that of Dybdal et al) [3]. Cooper et al used registers from both primary and secondary healthcare. Although they found a high HR for eating disorders (5.06; 95% CI 2.3, 10.9) in those with type 1 diabetes compared with those without diabetes, the confidence intervals were compatible with our findings as our upper limit of 3.09 is higher than their lower limit of 2.3 [4]. Due to our register-based setting focused solely on specialised healthcare, underdiagnosis of eating disorders is evident. Because records from primary healthcare were not included, it is likely that only the most severe eating disorders were detected in this study. As binge–purge spectrum symptoms are the most reported eating disorder symptoms in patients with type 1 diabetes [11], some eating disorder patients may not have been detected due to limitations of ICD-10 in detecting binge eating disorder. However, in Finland there has been a local agreement to diagnose binge eating disorder using ICD-10 codes F50.3, F50.8 or F50.9, which likely alleviates this problem to some degree. Our approach also missed any eating disorders that had not been detected in the healthcare system [43]. We are not aware of interview-based studies that would have estimated the incidence of eating disorders specifically among individuals with type 1 diabetes, but in general interview-based studies have found much higher estimates for the incidence of eating disorders than our register-based study [1, 44]. Nonetheless, despite underestimation of absolute incidence, the relative 2.35-fold higher incidence of eating disorders among those with type 1 diabetes compared with diabetes-free controls found in our study is supported by a large interview-based study, which found a 2.4-fold higher odds for eating disorders among people with type 1 diabetes compared with healthy control individuals [5].

We found that the higher incidence of eating disorders among individuals with type 1 diabetes vs diabetes-free control individuals was not explained by age, sex, socioeconomic status or place of residence. Previous studies have adjusted their models only for age and sex [3, 4]. Smaller studies have found that physical, psychological and family factors might increase the risk for disturbed eating among type 1 diabetes patients, yet they lacked comparison to diabetes-free participants [6–10]. To confirm these findings, replication in larger settings with comparison to diabetes-free control participants is needed. Furthermore, it has been unclear whether sex interacts with diabetes on the risk of incident eating disorders [3, 4]. Our finding of no interaction between type 1 diabetes and sex on the incidence of eating disorders indicates that type 1 diabetes comes with an elevated risk for eating disorders regardless of sex.

Our study comes with major strengths. Our nationwide, longitudinal setting with a diabetes-free control group and a large sample enabled us to expand earlier findings. Furthermore, our method of ascertaining psychotropic medication use is robust because we utilised registers that included only prescribed medications that had been purchased, increasing the probability that they had indeed been used. Finally, we had a sufficient number of male participants in our sample to assess sex differences.

Our study also has some weaknesses. First, we lacked information of clinically confirmed type 1 diabetes diagnoses. To identify type 1 diabetes patients, we used a literature-based method choosing insulin users who did not use any other glucose-lowering medications and were aged under 30 years [37, 38]. Based on earlier studies, this approach selects people with type 1 diabetes while excluding virtually all those with type 2 diabetes [37, 38], although there might be some severely ill type 2 diabetes patients whose poor renal function limits their diabetes medication to insulin only. Further, the few type 1 diabetes patients who were excluded by our methodology because their diabetes started after age 30 are not likely to have a major impact on our findings, because most eating disorders start in adolescence or early adulthood [45]. Second, we included both primary and secondary diagnoses of eating disorders in the analyses. This approach maximised the sensitivity of eating disorder ascertainment but might have biased our findings on eating disorder treatment. In some cases, eating disorder diagnoses might have been recorded as secondary diagnoses also in inpatient stays and outpatient visits where eating disorders were not the target of the treatment. However, our findings of eating disorder undertreatment are in line with previous studies, supporting the validity of our methods [25, 26].

In conclusion, we found over twofold higher incidence of eating disorders in individuals with type 1 diabetes compared with diabetes-free control individuals. The use of psychotropic medications among eating disorder patients was substantial and did not differ among those with vs without type 1 diabetes. However, those with type 1 diabetes received less outpatient hospital treatment for their eating disorders than those without diabetes. Our findings emphasise the need to reduce this discrepancy in treatment intensity in order to alleviate the burden of this severe dual condition of co-occurring eating disorder and type 1 diabetes.

## Supplementary Information

Below is the link to the electronic supplementary material.

ESM Table 1 (PDF 65 KB)

## Data Availability

The data used in this study are not publicly available due to restrictions applied to the availability of these data. However, data are available from the authors upon reasonable request and with permission of the Finnish Social and Health Data Permit Authority Findata.

## Abbreviations

aIRR: Adjusted incidence rate ratio
ATC: Anatomical Therapeutic Chemical
CARING: CAncer Risk and INsulin analoGues
IRR: Incidence rate ratio
