# Supplementary material for: Eating disorders among people with and without type 1 diabetes: incidence and treatment in a nationwide population-based cohort
Source: Diabetologia. 2025 Jan 4;68(4):766–77. doi: 10.1007/s00125-024-06346-7 (PMC11950106; doi:10.1007/s00125-024-06346-7)
Supplement: Supplementary file 1 — ESM Table 1 (PDF 65 KB) [file 125_2024_6346_MOESM1_ESM.pdf]

ESM Table 1. Distribution of eating disorder subtypes, *n* (%)

Eating disorder	All participants	Those with type 1 diabetes	Those with no diabetes
F50 (Any eating disorder)	250	175	75
F50.0 & F50.1 (Anorexia nervosa & Atypical anorexia nervosa)	81 (32.4)	51 (29.1)	30 (40.0)
F50.2 & F50.3 (Bulimia nervosa & Atypical bulimia nervosa)	51 (20.4)	34 (19.4)	17 (22.7)
F50.4, F50.5, F50.8, F50.9 & F50na[a]	118 (47.2)	90 (51.4)	28 (37.3)

[a]F50.4 (Overeating associated with other psychological disturbances); F50.5 (Vomiting associated with other psychological disturbances); F50.8 (Other eating disorders); F50.9 (Eating disorder, unspecified); F50na (eating disorder with no subtype classification).

χ^2 test for difference in the distribution of eating disorder subtypes between those with type 1 diabetes and those with no diabetes, pooled in 1) F50.0 and F50.1, 2) F50.2 and F50.3, and 3) all other types: $p = 0.111$.
